# Fine mapping and accurate prediction of complex traits using Bayesian Variable Selection models applied to biobank-size data

**DOI:** 10.1038/s41431-022-01135-5

**Published:** 2022-07-19

**Authors:** Gustavo de los Campos, Alexander Grueneberg, Scott Funkhouser, Paulino Pérez-Rodríguez, Anirban Samaddar

**Affiliations:** 1 Michigan State University, Department of Epidemiology & Biostatistics, East Lansing, MI USA; 2 Michigan State University, Department of Statistics & Probability, East Lansing, MI USA; 3 Michigan State University, Institute for Quantitative Health Sciences and Engineering, East Lansing, MI USA; 4 DNAnexus, Mountain View, CA USA; 5 Colegio de Postgraduados, Montecillo, Mexico

**Keywords:** Risk factors, Genome-wide association studies

## Abstract

Modern GWAS studies use an enormous sample size and ultra-high density SNP genotypes. These conditions reduce the mapping resolution of marginal association tests–the method most often used in GWAS. Multi-locus Bayesian Variable Selection (BVS) offers a one-stop solution for powerful and precise mapping of risk variants and polygenic risk score (PRS) prediction. We show (with an extensive simulation) that multi-locus BVS methods can achieve high power with a low false discovery rate and a much better mapping resolution than marginal association tests. We demonstrate the performance of BVS for mapping and PRS prediction using data from blood biomarkers from the UK-Biobank (~300,000 samples and ~5.5 million SNPs). The article is accompanied by open-source R-software that implement the methods used in the study and scales to biobank-sized data.

## Introduction

Genome-Wide Association Studies (GWAS) have reported large numbers of variants associated with many important traits and diseases; however, for complex traits many small-effect risk-loci remain unmapped. In the last decade, several public (e.g., UK-Biobank [1], Million Veteran Program [2], TOPMed, All of Us) and private (e.g., 23andMe^®^) initiatives have generated unprecedently large biomedical data sets comprising genotype data linked to extensive phenotype/disease data. These advances in data availability have not been fully matched with adequate changes in the analyses-methods used.

Single-marker-regression (SMR) remains the method most frequently used for mapping in GWAS. SMR tests for the marginal association between a phenotype (or a disease indicator) and individual SNPs and does not account for linkage disequilibrium (LD) between variants. Therefore, it can lead to significant associations of phenotypes with SNPs that are physically distant from causal variants–we refer to this phenomenon as poor mapping resolution. Importantly, the mapping resolution of SMR deteriorates with sample size because a large sample size increases the power to detect weak marginal associations between SNPs and phenotypes (Supplementary Data, Section 1). Therefore, for fine mapping, most genetic studies adopt some form of local variable selection approach to refine (SMR) GWAS-peaks to a smaller number of locally independent signals [3, 4]. However, these methods may reduce power due to cancellation of marginal effects (e.g., [5], this could happen if variants have effects with signs opposite to the sign of the covariance of the reference alleles at the two loci) and makes accurate error control challenging.

**Bayesian** variable selection (BVS) models [6, 7] offer a one-stop solution for fine mapping and Polygenic Risk Score (PRS) prediction, with the clear advantage that Bayesian models can provide accurate error control. However, the adoption of these methods in GWAS remained limited in part because achieving high power with these methods requires using a large sample size and because the computational burden of implementing BVS methods with ultra-high density SNP panels and biobank size data is substantial.

We implemented an efficient algorithm to generate samples from the posterior distribution of BVS models for problems involving hundreds of thousands of samples–the software is part of the BGLR R-package [8]. In this study, we use this software to study the power-FDR performance of BVS for mapping very small-effect risk loci. We compared the performance of a BVS method with a prior from the Spike-Slab (SS) family known as BayesC [9], with marginal-association testing (SMR), two other BVS methods, SuSiE [10] and FINEMAP [11], and two non-Bayesian variable selection procedures (LASSO, and a forward (FWD) regression). Furthermore, we used BayesC and SMR to map risk variants for six blood biomarkers related to metabolic syndrome. The empirical analysis shows that BayesC identifies most of the regions identified by SMR (and a many more) with a much finer mapping resolution than SMR.

## Materials and methods

We used data from the UK-Biobank [1] comprising genotypes and phenotypes of distantly related (pairwise genomic relationships smaller than 0.05) individuals of European background (*n* = 315,874). From the imputed genotype SNPs, after filtering (for a minor minor-allele-frequency >0.001 and a calling rate >0.95) and LD-pruning (R-squared <0.9), we retained 5,593,953 SNPs (see Supplementary Methods more details).

For the evaluation of power and FDR, we simulated complex traits with 500 (randomly chosen) casual variants and a trait heritability of 0.5 (i.e., on average a causal locus explained 1/10th of 1% of the phenotypic variance). We conducted 10 whole-genome simulations, each involving 500 causal loci and 5,593,453 SNPs without effects. We also considered a second simulation scenario with the same heritability and a smaller number of causal variants (50); thus, with larger SNP-effect sizes.

We evaluated six regression methods: marginal association testing (via SMR) and five variable selection methods (LASSO, FWD, and three Bayesian variable selection procedures). The SMR was a simple linear regression fitted via ordinary least squares using the phenotype as the response and one SNP as the predictor.

The *Variable Selection methods* were multiple regression models of the form

1 $$y = X\beta + \varepsilon$$

where $y = \left( {y_1,y_2, \ldots ,y_n} \right)\prime$ is a vector of phenotypes, $X = \left\{ {x_{ij}} \right\}$ is a matrix of genotypes, $\beta = \left( {\beta _1,\beta _2, \ldots ,\beta _p} \right)^\prime$ is a vector of SNP effects and $\varepsilon = \left( {\varepsilon _1,\varepsilon _2, \ldots ,\varepsilon _n} \right)^\prime$ is a vector of error terms.

### Local regressions

To apply variable selection methods on a whole-genome scale, we leveraged the fact that LD decays within relatively short distances; therefore, following Funkhouser et al. [12], we applied the variable selection method to overlapping segments containing 7000 contiguous SNPs (~4 Mbp for the imputed genotypes). This window of SNPs was displaced by 5000 SNPs, thus producing local regressions with a core of 3000 SNPs and flanking regions, each of ~2000 SNPs. From each regression we retrieved results from the core only (Supplementary Methods for more details).

The **LASSO** [13] regressions were fitted using the glmnet [14] R-package. The software produces a sequence of solutions $\{ {\hat \beta _{\lambda _1},\hat \beta _{\lambda _2}, \ldots .} \}$ over a grid of values of the regularization parameter (*λ*). We formed a grid with 1000 values that was evenly spaced in the log-scale. The same grid of values of *λ* was used across each of the segments to which LASSO regression was applied (see Local Regressions above). For each *λ* in the sequence we obtained a discovery set and a rejection set consisting of the SNPs with non-zero and zero effect in $\hat \beta _\lambda$, respectively. We ranked SNPs based on the value of *λ* at which the SNP becomes active in the model; these ranks were used to evaluate power and FDR over the regularization path.

The **Forward regression** also produces a sequence of solutions $\{ {\hat \beta _{FWD_1},\hat \beta _{FWD_1}, \ldots .} \}$ starting from the null model (no SNPs), then adding to the model one SNP at a time, at each step adding the SNP that produces the largest reduction in the residual sum of squares. The FWD regressions were applied to overlapping segments (see Local Regression above) and SNPs were ranked based on the reduction on the RSS produced when the SNP entered the model. These ranks were then used to evaluate power and FDR along the forward path.

For the **Bayesian Variable Selection** regression, we first used a model from the Spike-Slab family known as BayesC [9]. Briefly, the model assumes that the error terms in [1] are *iid* Normal $\varepsilon _i\sim ^{iid}N\left( {0,\sigma _\varepsilon ^2} \right)$; therefore, the conditional distribution of the data given the model parameters $\theta = \left\{ {\beta ,\sigma _\varepsilon ^2} \right\}$ was:

2 $$p\left( {y|\theta } \right) = MVN\left( {y|X\beta ,I\sigma _\varepsilon ^2} \right) \propto \left( {\sigma _\varepsilon ^2} \right)^{ - \frac{n}{2}}Exp\left\{ { - \frac{1}{{2\sigma _\varepsilon ^2}}\left( {y - X\beta } \right)^\prime \left( {y - X\beta } \right)} \right\}$$

where $MVN\left( {y|X\beta ,I\sigma _\varepsilon ^2} \right)$ represents a multivariate normal density with mean *Xβ* and (co)variance matrix $I\sigma _\varepsilon ^2$.

In a Bayesian models, priors that assign non-zero probabilities to null effects also specifies probabilities over possible models; this plays a very important role in error control [15]. Therefore, we consider a prior for SNP effects that has a point of mass at zero and a Gaussian slab

3 $$p\left( {\beta _j|\sigma _\beta ^2,\pi } \right) = \pi N\left( {\beta _j|0,\sigma _\beta ^2} \right) + \left( {1 - \pi } \right)1\left( {\beta _j = 0} \right)$$

where *π* $\left( {0 \le \pi \le 1} \right)$represents the proportion of loci with non-null effects and $\sigma _\beta ^2$ is the variance of effects (other common choices for the slab are the scaled-t and double-exponential). The prior used in BayesC [9] is equivalent to the one earlier proposed by George & McCulloch’s [16] with a Gaussian spike replaced with a point of mass at zero.

The hyper-parameters (*π*, $\sigma _b^2$ and $\sigma _\varepsilon ^2$) are unknown; thus, for the variance parameters we use scaled-inverse chi-square priors and for $\pi$ we use a Beta prior, $\pi \sim B\left( {\alpha _1,\alpha _2} \right)$ with $\alpha _1 = 1.1$ and $\alpha _1 = 99$, implying $E\left[ \pi \right] = 1.1/100$.

We compared the power-FDR performance of BayesC with that of SuSiE [17] and FINEMAP [11]. FINEMAP was developed to refine peaks detected in GWAS; therefore, we applied FINEMAP to segments detected through marginal association testing. The segments consisted of SNPs with single-marker-regression p-value smaller than 5e-8 that were at a distance of each other smaller than 1 Mbp. SuSiE was applied in a whole-genome scale using the same local regression approach used to implement BayesC.

### Bayesian FDR

We used the samples from the posterior distribution to estimate SNP-specific probabilities of association: $\pi _j = p\left( {\beta _j \,\ne\, 0|data} \right)$. The “local” FDR (LFDR [18]) for the *j*^*th*^ SNP with $\pi _j$ is simply $LFDR_j = 1 - \pi _j$. A decision rule that rejects $H_{0j}\,if\,\pi _j \, > \, \tau$ ($\tau \in [0,1]$) has an expected proportion of false discoveries equal to the average LFDR of the SNPs in the discovery set:

4 $$BFDR(\tau ) = 1 - \frac{1}{{p_\tau }}\mathop {\sum }\limits_{j:\pi _j > \tau }^{} \pi _j,$$

where *p*_*τ*_ is the number of SNPs in the discovery set. Expression [4] was evaluated for each SNP using the BFDR() function of the BGLR R-package [19].

### Software

SNP filtering was done using PLINK [20], genomic relationships were computed using the getG() function of the BGData R-package [21]. Single-marker regressions were performed using the GWAS() function of the BGData R-package. BayesC and SuSiE were implemented using the BGLR [19] (function BLRXy()) and susieR [17] R-packages, respectively. FINEMAP was fitted using the FINEMAP command line tool [11]. The *Forward regressions* were implemented using the FWD() function available in the BGData R-package, and LASSO regressions were fitted using the glmnet [14] R-package. Plots were generated using ggplot2 [22].

### Power and FDR determination

To estimate power-FDR curves, for each of the simulation scenarios and method we ranked SNPs based on the evidence for association produced by each method: (i) the p-values for the SMR (from smallest to larger), (ii) single-SNP posterior probabilities of inclusion for the BVS method (from largest to smallest, this was used for all the Bayesian models, (iii) the value of *λ* at which the SNP entered in the model for the LASSO regressions (from largest to smallest), and (iv) the reduction in the RSS produced when the SNP entered in the model in the FWD regression (from largest to smallest). We then produced discovery and rejection sets for each method by selecting the top-k SNPs of each of the ranks (k = 1, 2, …). For each discovery set we estimated the proportion of the 500 causal loci recovered in the discovery set and the proportion of SNPs in the discovery set that were not causal loci (i.e., the false discovery proportion).

To evaluate the ability of each method to fine-map causal variants we estimated the power-FDR performance at different mapping resolutions. Specifically, for an x-kbp mapping resolution (x = 10 kbp, 100 kbp, …, 1 Mbp), a discovery was considered true (false) if the distance with the closest causal variant was smaller (larger) than x-kbp.

### Analysis of six blood biomarkers

The simulation study demonstrated that the FWD and the BVS methods BayesC and SuSiE had the best performance. Furthermore, the performance of BayesC and SuSiE were very similar and better than that of FINEMAP; therefore, for analysis of the real data we used BayesC and SMR, which is the method most used in GWAS.

The biomarkers that we analyzed (glucose, serum urate (SU), serum creatinine, low- and high-density lipoprotein cholesterols (LDL and HDL, respectively), and triglycerides) are often monitored in medical checkups and are related to metabolic syndrome (see Table S1 of the Supplementary Data for sample size and descriptive statistics by trait).

Analyses were performed using the same genotypes used in the simulation (~5.6 million SNPs). All the traits were adjusted by the effects of sex, age, center, and with the top-10 SNP-derived eigenvectors. For rejection we used *p*-value < 5e-8 for the SMR and BFDR ≤0.05 or ≤0.10 for the BVS method. In regions of high-LD there may be multiple SNPs with elevated posterior probability of non-zero effect, with none of them reaching the single-SNP BFDR threshold (see Section I of the Supplementary Data for examples of this). Therefore, after identifying individual SNPs that cleared the BFDR thresholds mentioned above, we also identified short segments that had elevated inclusion probability but did not clear the BFDR-threshold. For these segments we estimated the posterior probability of the segment (i.e., the frequency at which at least one SNP from the segment was active in the model) and included that segment in the discovery set if the segment BFDR was smaller than 0.05 or 0.1. Therefore, the discovery sets for the BVS method consisted of individual SNPs and short segments that cleared one of the two BFDR thresholds.

### Polygenic risk scores

To evaluate the prediction accuracy of polygenic risk scores (PRS) we set aside data from 10,000 individuals for testing. As a baseline PRS we used one based on GWAS-significant SNPs (*p* value < 5e-8) with SNP effects estimated from SMR. These estimates do not account for LD; therefore, we considered a second PRS in which SNPs where selected based on SMR *p*-values and then SNP effects were estimated using BayesC. For these PRSs, we used p-value thresholds for SNP selection ranging from 1e-12 to 1e-4. Finally, we considered a whole-genome PRS derived using the estimates of effects from the local Bayesian regressions implemented using model BayesC (the same approach used for mapping). These local Bayesian regressions covered all the available SNPs (~5.6 million); however, to simplify the computation of the PRS we only used the SNPs with posterior inclusion probability greater than 1/1000.

## Results

**Fig. 1:**
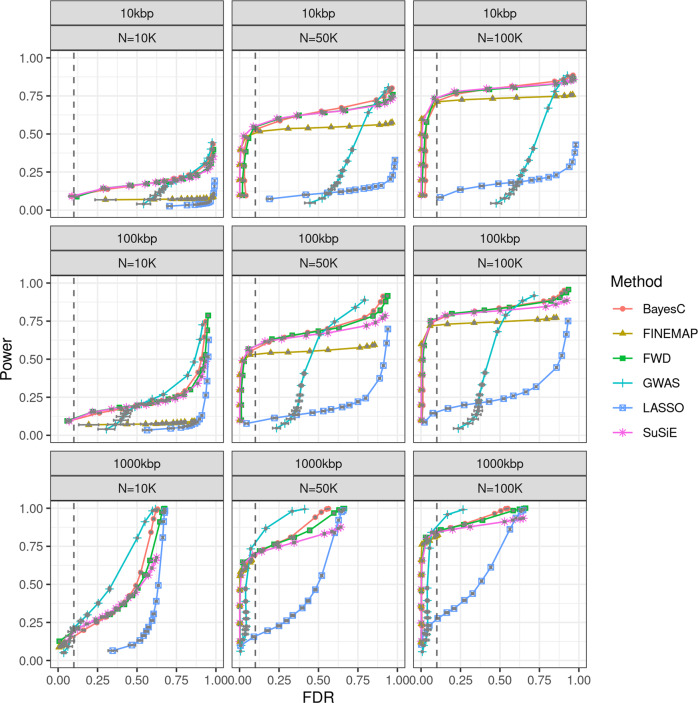
Power-FDR (False Discovery Rate) curves by sample size, mapping resolution, and statistical method used. For a mapping resolution of x-kbp, a SNP in a discovery set was considered a true discovery if its distance to the closest simulated causal variant was closer than x-kbp.

The power-FDR curves estimated from the simulation scenario with heritability 0.5 and 500 causal loci are displayed in Fig. 1 (and File S1 of the Supplementary Data). For a sample size of 10,000 and a mapping resolution of 100 kbp (top-left panel of Fig. 1) all the methods had relatively low power–this was expected because individual SNPs with non-null effect explained only 1/1000 of the phenotypic variance. Increasing sample size improved the power-FDR performance of all the methods; however, the variable selection methods improved their performance much more than the SMR. Among the variable selection procedures, the BVS methods (including BayesC, SuSiE, and FINEMAP) and the FWD regression were the best performing ones. Importantly, with a large sample size these methods had a very sharp phase-transition in the power-FDR curve showing that, with a large sample size, both methods can achieve high power with very low FDR even for very small effect variants. This was evident even with a mapping resolution of 10 kbp (see top-right plot in Fig. 1). On the other hand, the SMR only achieve a comparable power-FDR performance with a mapping resolution of 1 Mbp (see lower-right plot) demonstrating that with a large sample size mapping based on SMR *p*-values produces a large proportion of discoveries that are more than 100 kbp apart from the causal variants. Among the Bayesian methods, SuSiE and BayesC performed very similarly and FINEMAP had a slightly lower power for an FDR of 0.1 (see Fig. 1, top two panels for sample size 50,000 and 100,000). This small reduction in power may result from some of the small-effect causal variants not reaching GWAS-significant values; thus, not making it to the second step.

The results from the simulation scenario with larger effect sizes (heritability 0.5, 50 causal variants, Fig. S4) were similar to the ones obtained in the simulation scenario with 500 causal variants in that FWD, SuSiE, and BayesC achieved the best power-FDR performance and had very sharp power-FDR transitions. However, as expected, for any given sample size and FDR in this scenario these three methods achieved higher power than in the scenario with smaller effect sizes (500 causal variants). On the other hand, the power-FDR performance of SMR was worst in the scenario with larger effects (50 causal variants) than in the scenario with smaller effects. This happens because large effect loci can generate marginal association significant results even for a very weak LD (i.e., at a long physical distance) between the marker and the causal variant.

### Bayesian FDR-control

**Fig. 2:**
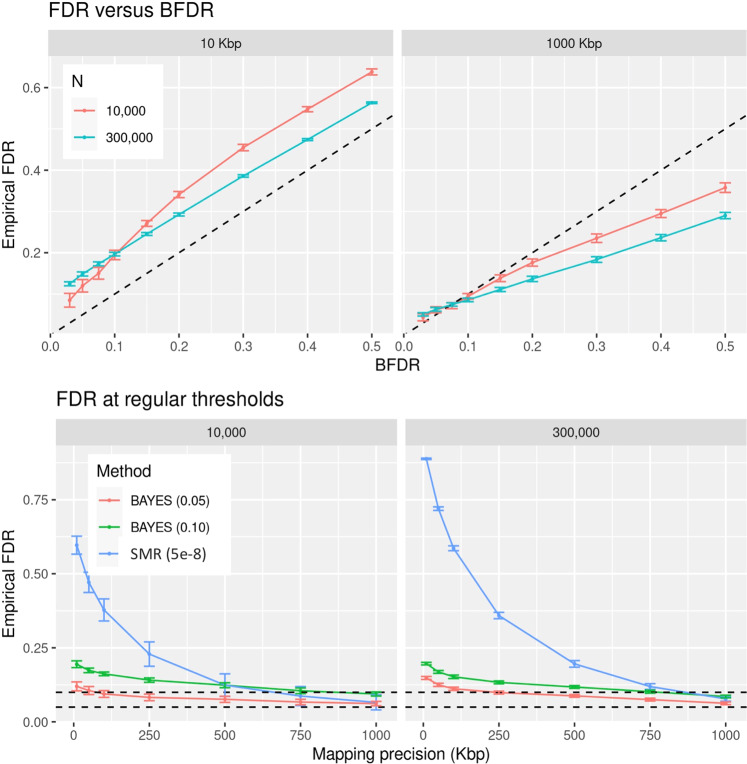
Empirical False Discovery Rate (FDR) by decision rule, sample size, and mapping resolution. Top panel: Empirical FDR versus Bayesian FDR threshold used to determine significance, by sample size. Bottom panel: Empirical FDR by mapping resolution for three decision rules: SMR *p* value < 5e-8, BFDR < 0.05 and BFDR < 0.1. All the results in this figure are based on the simulation scenario involving a heritability of 0.5 and 500 causal variants. For decision rules using BFDR, results were obtained using model BayesC. For a mapping resolution of x-kbp, a SNP in a discovery set was considered a true discovery if its distance to the closest simulated causal variant was closer than x-kbp.

We used the results from the most challenging simulation scenario (heritability 0.5, 500 causal variants) to evaluate the empirical FDR of standard decision rules including SMR *p*-value ≤ 5e-8 and BFDR ≤ 0.10 or 0.05. The results are summarized in Fig. 2, Figs. S5, S6. For a 1 Mbp mapping resolution the standard rule used in GWAS SMR *p*-value ≤5e-8 leads to an FDR of ~0.08, comparable to a decision rule using BFDR ≤ 0.1, and a bit higher than using BFDR ≤ 0.05 (lower panel of Figs. 2 and S5, S6). However, for finer mapping resolutions (e.g., 125 kbp) a decision rule rejects if SMR *p*-value ≤ 5e-8 can produce a rate of false discoveries greater than 50%. Importantly, for the SMR, the exponential growth of the FDR with increasingly finer mapping resolution was more marked with large sample size, illustrating once again how the mapping resolution of SMR deteriorates with sample size. On the other hand, while the BVS model also had an increasing FDR with finer mapping resolution, the slope of the curves was very small compared with that of the SMR suggesting that the prior provide reasonably effective (albeit not perfect) error control. We conclude from these results that, for data from unrelated white Europeans, using a BFDR < 0.05 as a decision rule leads to an FDR ≤ 0.1 for a mapping resolution of ~125 kbp.

### High resolution mapping of risk loci associated with six metabolic syndrome-associated blood biomarkers

**Fig. 3:**
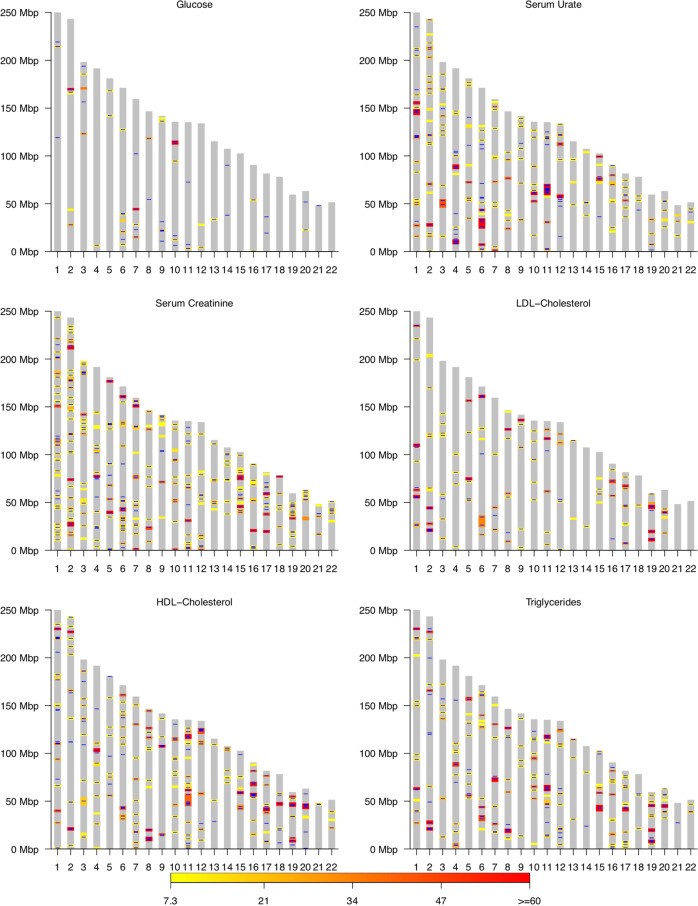
Regions assciated to each of the six blood biomarkers studied. Ideogram displaying segments identified through single-marker regression (red-yellow bands corresponding to -log10(pvalues)) and by a Bayesian Variable Selection (BayesC) model (blue lines correspond to variants and segments with BFDR < 0.1).

Table 1 and Fig. 3 display the results of the SMR and of BayesC. The number of variants with SMR-significant marginal association ranged from 469 (Glucose) to 5991 (serum urate). We grouped the SMR-significant variants into non-overlapping chromosome segments, each including all the SMR-significant variants that were at a distance smaller than 1000 Mbp. The number of segments harboring SMR-significant variants ranged from 43 (Glucose) to 225 (HDL-Cholesterol); these regions are displayed in yellow-red scale in Fig. 3.

**Table 1:** Number of independent segments discovered (and the number of SNPs included in those segments) by method and overlap between them.

	Discoveries			Overlap^c^	
	BayesC^a^		SMR^b^		
	BFDR ≤ 0.05	BFDR ≤ 0.10		BFDR ≤ 0.05	BFDR ≤ 0.10
Glucose	41 (46)	54 (60)	43 (469)	67.4%	76.7%
Serum Urate	194 (216)	244 (264)	175 (5991)	69.1%	81.1%
Serum Creatinine	228 (264)	296 (331)	225 (4394)	75.1%	84.9%
HDL-Cholesterol	246 (274)	307 (330)	177 (5909)	77.4%	86.4%
LDL-Cholesterol	129 (139)	161 (168)	99 (3802)	77.8%	87.9%
Triglycerides	200 (213)	246 (264)	158 (5679)	71.5%	84.2%

^a^Total number of discoveries, in between parenthesis the number of discoveries that were single-SNPs clearing the Bayesian FDR (BFDR) threshold.

^b^To map individual variants into chromosome segments, we merged all the discoveries (SNPs with *p*-value < 5e-8) that were at a 1000 kbp or shorter distance of each other.

^c^% of the segments detected by SMR that had at least one Bayesian discovery inside the segment.

BayesC identified a much smaller number of variants than the SMR; however, the number of independent segments identified by BayesC were typically higher than those identified by SMR except for Glucose. Most often BayesC selected one or a few variants within each of the segments (Fig. 3). The segments identified by BayesC were often very short–the median length was about 30 kbp–36 kbp. On the other hand, the SMR-segments had a median length of 142.5 kbp.

### Polygenic prediction

**Fig. 4:**
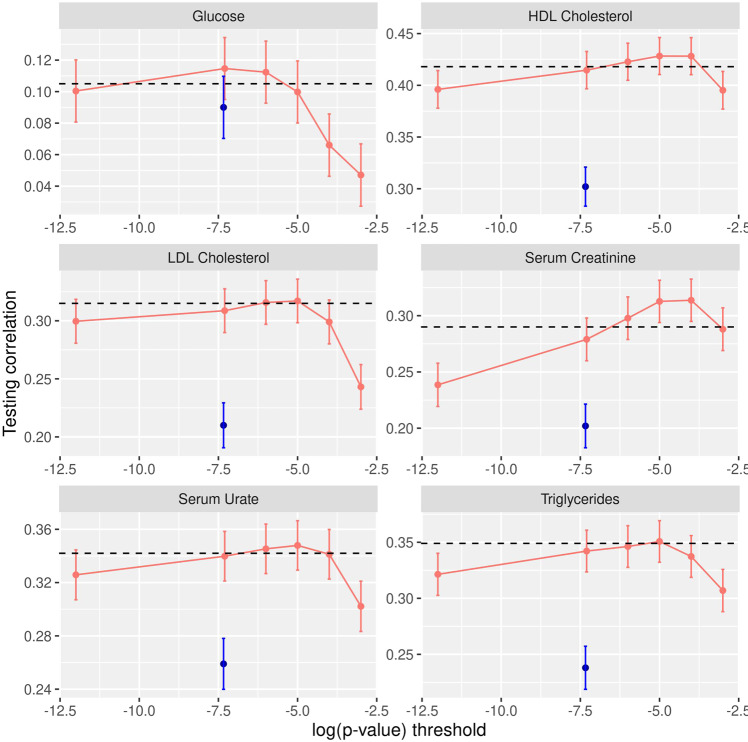
Prediction correlation in testing set for various polygenic risk scores. The blue dots are the prediction correlations obtained with GWAS-significant SNPs (*p*-value < 5e-8) and SNP effects estimated from single-marker regressions (SMR). The pink-salmon curve shows the prediction accuracy of sets of SNPs selected using the log10(p-value) threshold given in the horizontal axis, with SNP effects estimated using BayesC. The horizontal dashed black line gives the prediction accuracy of a whole-genome Bayesian regression (BayesC) applied using overlapping local regressions.

Figure 4 and Table S2 show the prediction correlations obtained in testing sets. A PRS based on GWAS-significant SNPs (SMR *p*-value < 5e-8) and with SNP effects estimated from SMRs achieved prediction correlations ranging from 0.09 (+/− 0.01, Glucose) to 0.302 (+/− 0.01, HDL Cholesterol)–the results from these PRSs are represented in blue in Fig. 4 (see also Table S2). The estimates of effects from SMR do not account for LD; re-estimating the SNP effects of GWAS-significant SNPs using BayesC led to significant increases in prediction correlations. The gains in prediction correlation achieved by re-estimating the effects of GWAS-significant SNPs using BayesC ranged from 17% (glucose) to 47% (triglycerides). The PRS that used the estimates of effects from the whole-genome Bayesian regressions (horizontal dashed black lines in Fig. 4, see also Table S2) were very similar to the ones obtained by a PRS based on GWAS-significant SNPs with effect estimates derived using BayesC. Furthermore, for all traits but creatinine, the prediction accuracy achieved by the whole-genome Bayesian regression were within the margin of error of the maximum prediction accuracy that one could obtain in this data set by selecting SNPs using *p*-values from SMR and then estimating the effects of the SNPs using BayesC (i.e., the maximum of the salmon curve in Fig. 4).

## Discussion

Modern genetic studies use a very large sample size and ultra-high-density genotypes (potentially millions of SNPs). In principle, the large sample size and the high-marker density should improve our ability to map risk variants. However, these conditions deteriorate the mapping resolution of SMR–the most frequently used methodology used in GWAS. We illustrated this problem with extensive simulations and with the analysis of six blood biomarkers. With a sample size of ~300,000 and high marker density, SMR can lead to significant associations for variants that are up to 300–1000 kbp apart from the causal variant depending on the effect size, and the extent of LD in the region (Figs. S1, S2). This results in poor power-FDR performance (Fig. 1, Fig. S4); thus, when marginal association testing is applied to biobank-size data and ultra-high-density genotypes, high power can only be achieved at the price of a very high FDR.

To address the poor mapping resolution of SMR several methods have been proposed. One approach is to ‘weight’ the evidence of association of SNPs within a region to estimate an approximate posterior probability of association [3, 23]. However, this approach assumes that only one SNP (in the region) has an effect and do not fully account of multi-locus LD in the region. Another common approach is to use two-steps procedures in which first a marginal-association test is used to identify chromosome segments harboring GWAS-significant variants and then, in a second step, the GWAS-summary statistics obtained in the first step are used, in conjunction with an LD-reference panel, to identify independent signals. However, in the first step the procedure may miss important signals due to “unfaithfulness” or cancellation of marginal effects [5]. Additionally, the use of a reference panel to approximate LD patterns may not accurately reflect the LD-patterns of the data set used to derive the GWAS summary statistics in the first place. The slightly worse performance of FINEMAP is likely reflecting a loss of power due to the use of a 2-step procedure. Furthermore, we note that our results are likely giving an optimistic view of the performance of two step procedures because, here, the LD-matrix was computed using the same data set that was used to obtain the SMR summary statistics. If, as often done, the LD-matrix is computed from a reference panel (with possibly different LD patterns than the data set used to derive the summary statistics) the loss of power may be higher.

To address limitations of two-steps procedures, here we considered four variable selection methods (FWD, LASSO, and two variable selection procedures: BayesC and SuSiE priors) that account for multi-locus LD. These methods are not new; however, the adoption of these methods in human GWAS has been limited in part because achieving high power with variable selection methods often requires a very large sample size. The advent of Big Data in genomic research has opened new opportunities for the use of these methods in GWAS.

Among the four variable selection methods considered, the FWD regression and the BVS methods (both SuSiE and BayesC) were the ones that achieved the best power-FDR performances. With a large sample size (*n* ≥ 100,000) these two methods can achieve high power with low FDR and very fine mapping resolution, even for very-small-effect variants.

BayesC, a Bayesian method with a Spike-Slab prior, and the FWD regression achieved a very good (and remarkably similar) power-FDR performance. This is not surprising considering the links that exist between these two methods and subset selection. The FWD regression is an approach developed to approximate subset selection constraining the search to a path that adds one predictor at a time [24]. Furthermore, the objective function of subset selection, $\hat \beta = argmin\left\{ {RSS\left( {y,X,\beta } \right) + \lambda \Sigma _j1\left( {\beta _j \,\ne\, 0} \right)} \right\},$ can be seen as the logarithm of the kernel of the posterior distribution of a Bayesian model with a Gaussian likelihood and a prior on SNP effects with a point of mass and a flat slab, which is similar to the prior used in BayesC.

Collecting samples from the posterior distribution of high dimensional Bayesian models is computationally demanding. However, advances in hardware and in algorithms has made the application of BVS to biobank-size data feasible. As a reference we provide in Supplementary Fig. S7 the estimated computing time required for BLRXy() to generate 10,000 posterior samples as a function of number of SNPs in the model (from 1000 to 10,000 SNPs) and sample size (we evaluated up to *n* = 300,000). The information in the appendix also provides the computing times required for up to 100 iterations of SuSiE and SuSiE-sufficient statistics. It took on average 17 min for BLRXy() to generate 10,000 posterior samples for a model involving 10,000 SNPs and a sample size of 300,000. The computing times of BLRXy() were similar to those of SuSiE-sufficient statistics and considerably lower than those of SuSiE when sample size was large. These results show that it is doable to apply Bayesian regressions using the local-overlapping segments approach used in Funkhouser et al. [12] and adopted here.

In this study we focused on a specific BVS model that uses a prior with point of mass at zero and a Gaussian slab. Our simulation results suggest that the power-FDR performance of different BVS methods (e.g., BayesC, SuSiE) is very similar (see Fig. 1) provided that the prior induces some form of variable selection. There are many other variable selection priors that we anticipate will perform similarly, including priors from the spike slab family that use non-Gaussian slabs (e.g., scaled-t [25], or double-exponential [26, 27]).

One concern that is often raised about Bayesian models is the need of specifying prior hyper-parameters and the influences that these may have on inferences. In the case of BayesC there are two hyper-parameters: the prior proportion of non-zero effects and the variance of the slab. To avoid specifying these hyper-parameters a-priori, we treated them as unknown and assigned priors to each of them. For the variance, we choose a scaled-inverse chi-square with small DF which results in limited influence of the prior on inferences when sample size is large. For the proportion of non-zero effects, we used a Beta prior with a prior mean of 1/100 (i.e., assuming a prior that 1% of the SNPs have none-zero effect). One could use a uniform prior (which is a special case of the Beta); however, adequate FDR control and stringent variable selection can be better achieved by using priors that are informative; this can be particularly important for studies involving a much smaller sample size than the one presented here.

In regions of high LD collinearity may lead to many SNPs with elevated inclusion probability without any of them reaching stringent FDR thresholds (e.g., BFDR < 0.1); thus, reducing power. In our analysis of blood biomarkers, we illustrated how this problem can be addressed using methods which identify sets of variants that are jointly associated with a phenotype.

Finally, we evaluated various strategies to build PRSs; our results suggest that the prediction accuracy that can be achieved using a whole-genome BVS procedure implemented using local regressions is similar to the highest prediction accuracy that can be achieved fitting a BVS to SNPs filtered based on marginal association tests. Therefore, we conclude that BVS applied using local Bayesian regressions can be used for both fine mapping and accurate PRS prediction.

## Supplementary information

Supplementary Methods

Supplementary Data

## Data Availability

The data that supports the findings of this study are available from the UK-Biobank but restrictions apply to the availability of these data, which were used under license for the current study, and so are not publicly available. Data are however available from the authors upon reasonable request and with permission of the UK-Biobank.

## References

[1] Sudlow C, Gallacher J, Allen N, Beral V, Burton P, Danesh J (2015). UK Biobank: an open access resource for identifying the causes of a wide range of complex diseases of middle and old age. PLOS Med [Internet].

[2] Gaziano JM, Concato J, Brophy M, Fiore L, Pyarajan S, Breeling J, et al. Million Veteran Program: A mega-biobank to study genetic influences on health and disease. J Clin Epidemiol [Internet]. 2016 Feb 1 [cited 2018 Mar 31];70:214–23. Available from: http://linkinghub.elsevier.com/retrieve/pii/S0895435615004448. 10.1016/j.jclinepi.2015.09.016 26441289

[3] Mahajan A, Taliun D, Thurner M, Robertson NR, Torres JM, Rayner NW (2018). Fine-mapping type 2 diabetes loci to single-variant resolution using high-density imputation and islet-specific epigenome maps. Nat Genet [Internet].

[4] Yang J, Ferreira T, Morris AP, Medland SE, Madden PAF, Heath AC (2012). Conditional and joint multiple-SNP analysis of GWAS summary statistics identifies additional variants influencing complex traits. Nat Genet.

[5] Wasserman L, Roeder K (2009). High-dimensional variable selection. Ann Stat [Internet].

[6] George EI, McCulloch RE (1993). Variable selection via Gibbs sampling. J Am Stat Assoc [Internet].

[7] Ishwaran H, Rao JS. Spike and slab variable selection: Frequentist and bayesian strategies. Vol. 33, Annals of Statistics. Institute of Mathematical Statistics; 2005. p. 730–73.

[8] Pérez P, de los Campos G (2014). Genome-wide regression and prediction with the BGLR statistical package. Genet [Internet].

[9] Habier D, Fernando R, Kizilkaya K, Garrik DJ. Extension of the {B}ayesian Alphabet for Genomic Selection. BMC Bioinformatics. 2011;12. 10.1186/1471-2105-12-186 PMC3144464 21605355

[10] Wang G, Sarkar A, Carbonetto P, Stephens M. A simple new approach to variable selection in regression, with application to genetic fine mapping. J R Stat Soc Ser B Statistical Methodol [Internet]. 2020;82:1273–300. https://rss.onlinelibrary.wiley.com/doi/abs/10.1111/rssb.12388. 10.1111/rssb.12388 PMC10201948 37220626

[11] Benner C, Spencer CCA, Havulinna AS, Salomaa V, Ripatti S, Pirinen M (2016). FINEMAP: efficient variable selection using summary data from genome-wide association studies. Bioinforma [Internet].

[12] Funkhouser SA, Vazquez AI, Steibel JP, Ernst CW, Campos G de los. Deciphering sex-specific genetic architectures using local Bayesian regressions. bioRxiv [Internet]. 2019 May 31 [cited 2019 Jun 15];653386. Available from: https://www.biorxiv.org/content/10.1101/653386v1. 10.1534/genetics.120.303120 PMC7198271 32198180

[13] Tibshirani R (1996). Regression shrinkage and selection via the {LASSO}. J R Stat Soc Ser B.

[14] Friedman J, Hastie T, Tibshirani R (2010). Regularization paths for generalized linear models via coordinate descent. J Stat Softw [Internet].

[15] Scott JG, Berger JO (2010). Bayes and empirical-Bayes multiplicity adjustment in the variable-selection problem. Ann Stat [Internet].

[16] George EI, McCulloch RE (1993). Variable Selection via {G}ibbs sampling. J Am Stat Assoc.

[17] Wang G, Sarkar A, Carbonetto P, Stephens M. A simple new approach to variable selection in regression, with application to genetic fine mapping. J R Stat Soc Ser B Statistical Methodol [Internet]. 2020;82:1273–300. https://onlinelibrary.wiley.com/doi/10.1111/rssb.12388. 10.1111/rssb.12388 PMC10201948 37220626

[18] Efron B, Hastie T. Computer Age Statistical Inference. Cambridge University Press; 2016.

[19] Pérez P, De Los Campos G. Genome-wide regression and prediction with the BGLR statistical package. Genetics. 2014;198. 10.1534/genetics.114.164442 PMC4196607 25009151

[20] Chang CC, Chow CC, Tellier LC, Vattikuti S, Purcell SM, Lee JJ (2015). Second-generation PLINK: rising to the challenge of larger and richer datasets. Gigascience [Internet].

[21] Grueneberg A, de Los Campos G BGData - A Suite of R Packages for Genomic Analysis with Big Data. G3 (Bethesda) [Internet]. 2019 May 7 [cited 2019 Jul 10];9:1377–83. Available from: http://www.ncbi.nlm.nih.gov/pubmed/30894453. 10.1534/g3.119.400018 PMC6505159 30894453

[22] Wickham H ggplot2: Elegant Graphics for Data Analysis [Internet]. Springer-Verlag New York; 2016. Available from: https://ggplot2.tidyverse.org.

[23] Maller JB, McVean G, Byrnes J, Vukcevic D, Palin K, Su Z (2012). Bayesian refinement of association signals for 14 loci in 3 common diseases. Nat Genet [Internet].

[24] Draper NR, Smith H. Applied regression analysis. Applied Regression Analysis. wiley; 2014. 1–716 p.

[25] Meuwissen THE, Hayes BJ, Goddard ME (2001). Prediction of total genetic value using genome-wide dense marker maps. Genetics..

[26] Park T, Casella G (2008). The {B}ayesian {LASSO}. J Am Stat Assoc.

[27] de los Campos G, Naya H, Gianola D, Crossa J, Legarra A, Manfredi E (2009). Predicting quantitative traits with regression models for dense molecular markers and pedigree. Genetics..

