# Supplementary material for: Fine mapping and accurate prediction of complex traits using Bayesian Variable Selection models applied to biobank-size data
Source: Eur J Hum Genet. 2022 Jul 19;31(3):313–20. doi: 10.1038/s41431-022-01135-5 (PMC9995454; doi:10.1038/s41431-022-01135-5)
Supplement: Supplementary file 1 — Supplementary Methods [file 41431_2022_1135_MOESM1_ESM.docx]

**Supplementary Methods**

**Sample selection**

The genotypes of UK-Biobank participants of White British ancestry from the UK Biobank that have not withdrawn (n = 409,629) were used to compute a genomic relationship matrix (more below). We use this to identify 315,874 distantly related individuals (using a cutoff of 0.05 for the genomic relationship); the simulation study used genotypes of randomly selected individuals (from n=10,000 to n=300,000) from that set. After excluding samples with missing values for each trait, the number of samples with genotype and phenotype data were: 276,708 (glucose), 301,376 (serum urate), 301,594 (serum creatinine), 301,223 (LDL-cholesterol), 276,887 (HDL-cholesterol), and 301,508 (triglycerides). For each trait, 10,000 of these samples were saved for the assessment of prediction accuracy. These samples were not used in any of the model building steps.

**Genotypes**

We used SNPs from the imputed UK Biobank panel (93,095,623 SNPs before data edits). From this set, we removed variants with minor-allele frequency <0.1% and a missing call rate of >5%. We also edited out SNPs mapped to the same position. Finally, we used PLINK to LD-prune the imputed SNPs using an R-squared threshold of 0.9, a window size of 200 SNPs, and a frame shift of 50 SNPs. The number of SNPs retained after these data edits was 5,593,953.

**Analysis of biobank-sized data in the R-environment**

All the software used in this study runs in the R-environment. With the sample size (n~300,000) and number of SNPs (p~5.5 million) considered in this study loading genotypes in memory is not feasible, at least with commodity hardware. To circumvent this problem, we used memory mapping tools (BEDMatrix) and the linked arrays (LinkedMatrix) implemented in the BGData R-package (21). These classes and methods allow performing computations on segments of the files (subsets of the rows and columns) in memory without loading the entire genotype data set.

**Genomic relationships**

We used the getG() function of the BGData R-package (21) to compute additive genomic relationships among individuals of European ancestry, and then used the findRelated() function of the same package to identify a set of distantly related individuals, defined as pairs with a genomic relationship smaller than 0.05.

**Simulated phenotypes**

For power-FDR analysis we simulated highly complex traits with 500 randomly chosen loci from the UK-Biobank imputed SNPs. We partitioned the genome into 1Mpb segments, sampled 500 segments and chose the position of the causal variant at random within the segment. SNP effects were sampled from a normal distribution, with the variance of effects and the error variances adjusted to match a trait heritability of 0.5. Therefore, on average each SNP explained 1/10^th^-of-1% of the phenotypic variance. In a second scenario, we used 50 causal loci and a heritability of 0.5; thus, in this scenario, on average, each loci explained 1% of the phenotypic variance.

**Local Regressions**

Within each chromosome we applied the variable selection procedures to 7,000 consecutive SNPs. We displaced these windows by 2,000 SNPs, thus producing overlapping segments, with cores consisting of 3,000 SNPs, and flanking regions of 2,000 on each of the flanks. From each of the models that were fitted, we used the results produced for the 3,000 SNPs in the core and discarded the results for the SNPs in the flanking regions which were used to account of SNPs in neighboring segments (possibly in LD with those in the core). The process is represented graphically for a conceptual example involving 9,000 SNPs. Analyzing these 9,000 SNPs using the approach described above requires fitting three models (Fit 1 through Fit 3 below), each time retrieving the results from the cores only.

**Marginal association analysis**

Single-marker regression analysis were based on ordinary least square regression of the simulated or adjusted phenotypes on each SNP, one-SNP-at-a-time. These analyses were done using the GWAS() function of the BGData R-package (21).

**Software for variable selection methods**

Documentation for the glmnet R-package (used here to fit LASSO regressions) can be found in the publication that introduced the software (14) and in vignettes linked to the package.

Model BayesC was fitted using the BGLR R-package (18). This package offers three functions to fit shrinkage/variable selection models: BGLR(), BLRXy(), and Multitrait(). The BGLR() and BLRXy() functions fit single-trait models, BGLR() is optimized for problems with *p* (number of SNPs) greater than sample size. To achieve high computational efficiency for problems where sample size is larger than the number of SNPs fitted, the BLRXy() function of the BGLR R-package first computes sufficient statistics ($X'X$ and $X^{'}y$) from the genotype ($X$) and phenotype ($y$) and then generates samples from the posterior distribution of the BVS model using Gibbs sampling. This makes the computational time independent of sample size, once the summary statistics were computed. Examples on how to fit models using this function can be found in the GitHub repository for BGLR. We used the estimated posterior probabilities of non-zero effect (and the corresponding BFDR) to rank SNPs for power and FDR determination (more below).

The forward regressions were fitted using the FWD() function of the BGData R-package (21). Like BLRXy(), FWD() first computes sufficient statistics ($X'X$ and $X^{'}y$) from the genotype ($X$) and phenotype ($y$) and then solves the FWD regression problem adding to the model each time the SNP that produces the largest residual sum of squares to the model, which can be derived from the sufficient statistics. FWD returns the forward path (what SNP entered in each step as well as least-square estimates of effects, the residual sum of squares, AIC, and BIC for each of the models in the path.

SuSiE was fitted using the function susie_suff_stat() function of susieR R-package (19). This function fits model using sufficient statistics $X'X$ and $X^{'}y$ which we computed from (centered) genotypes and pre-adjusted phenotypes. We applied SuSiE in a whole-genome scale, using the same local-Bayesian-regression setting used for BayesC (see Local Regressions, above).

Finally, we considered using FINEMAP (11) to refine findings from regions harboring GWAS-significant associations. To this end, we identify from the results of the SMR analysis regions harboring SNPs that had association p-values < 5e-8 and were at a distance smaller than 1Mbp (these were found using the segments() function of the BGData R-package). Then we applied FINEMAP to each of the segments identified and retrieved from each of these analyses the estimated posterior probability of inclusion reported by FINEMAP.

**Blood biomarker data**

Data for the six blood biomarkers (glucose, serum urate, serum creatinine, LDL-cholesterol, HDL-cholesterol, and triglycerides) and covariate information was obtained from the first visit, if the phenotype was missing at the first visit, data was retrieved from follow-up visits. After inspecting the histograms for each trait, we decided to log-transformed creatinine, HDL-cholesterol, triglycerides, and serum urate to achieve a reasonably symmetric distribution. All the traits were pre-adjusted by sex, center, age, and 10 SNP-derived principal components.

**Post-processing of GWAS results**

To report results for the six blood biomarkers we grouped the discoveries from the SMR and from the BVS into non-overlapping genomic regions using the segments() function of the BGData R-package. This function takes a set of findings, a map, and a base-pair distance threshold and groups into segments all the findings that are at a distance smaller or equal than the specified threshold.

Most plots were done using ggplot2 (22) The ideogram of Figure 3 was produced using the explore() function of the GWASExplorer R-package, available at <https://github.com/QuantGen/GWASExplorer>. This function produces an interactive plot that the user can use to zoom into regions, producing plots such as the ones presented in Figure 3.
