# Supplementary material for: Fine mapping and accurate prediction of complex traits using Bayesian Variable Selection models applied to biobank-size data
Source: Eur J Hum Genet. 2022 Jul 19;31(3):313–20. doi: 10.1038/s41431-022-01135-5 (PMC9995454; doi:10.1038/s41431-022-01135-5)
Supplement: Supplementary file 2 — Supplementary Data [file 41431_2022_1135_MOESM2_ESM.docx]

**Supplementary Data**

**I. An illustration of the effect of sample size on marginal association tests and on the Bayesian Variable Selection (BVS) procedure**

We present three stylized simulation examples to illustrate how a large sample size affects the mapping resolution of single-marker regression (SMR) and of the BVS procedure. The examples used genotypes from three regions:

- chromosome 1, mega-base-pair (Mbp) position 10-11,

- chromosome 4, Mbp position 8.5-11.5 (this region harbors the SLC2A9 gene, a glucose transporter that has been associated with serum urate levels), and

- chromosome 16, Mbp position 53.5 to 54.5 (this region harbors the FTO gene).

For each of these regions, we simulated a phenotype that had two causal variants located in the center of each region. Each causal variant explained 0.5% of the trait variance. Subsequently, we tested the association of the phenotype with all the SNPs in the region plus those in each the 1 Mbp flanking regions using single-marker regression and a Bayesian Variable Selection (BVS) procedure. We conducted these analyses with data from the UK Biobank using a sample size of n=10,000 (10K) and n=300,000 (300K).

The results of these stylized examples are presented in Figures S1 through S3. With a sample size of 10K the SMR identified the region harboring the two causal variants with a relatively good mapping resolution, that is, with this sample size there were not many SNPs outside of the region harboring the two causal variants with significant association. However, when the sample size was increased to 300K, the mapping resolution deteriorated markedly, with significant hits as far as 1 Mbp apart from the causal variants (e.g., lower-left panel of Figure S3). On the other hand, with a sample size of 10K the BVS method identifies the causal variants and nearby SNPs having elevated posterior probability of non-zero effect with a precision comparable to that of the SMR. However, when we increased the sample size to 300K the BVS procedure clearly identifies the two causal variants from all the other variants in the region. In five of the six causal variants simulated (Figures S1 and S3, and the right-causal variant in Figure S2) the causal variants achieve very high posterior probability of non-zero effect; however, in one of the examples (left causal variant of Figure S2) there were two SNPs (which are in high LD) each with posterior probability of association close to 0.5 each; however the joint posterior probability of association of these two SNPs, $p\left( \beta_{j}\neq0 or \beta_{j^{'}}\neq0|data \right)$, was very close to 1. These examples illustrate three important concepts that motivate our study: (1) The mapping resolution of marginal association tests deteriorate with sample size, (2) Achieving high power with variable selection procedures requires using a large sample size, (3) In regions of high LD, the BVS may identify sets of SNPs (and not individual variants) that are jointly associated with a phenotype.

**Figure S1**: Mapping risk loci using single-marker regression (left) and a Bayesian Variable Selection procedure (right) with moderate (N=10K, K=1,000) and very large (N=300K) sample size. In the simulation there were two causal variants (located in chromosome 1, Mbp positions 9 through 12), each explaining 0.5% of the simulated phenotype.

**Figure S2:** Mapping risk loci using single-marker regression (left) and Bayesian Variable Selection procedure (right) with moderate (N=10K, K=1,000) and very large (N=300K) sample size. In the simulation there were two causal variants (located in chromosome 4, Mbp positions 8.5 through 11.5), each explaining 0.5% of the simulated phenotype.

**Figure S3**: Mapping risk loci using single-marker regression (left) and Bayesian Variable Selection procedure (right) with moderate (N=10K, K=1,000) and very large (N=300K) sample size. In the simulation there were two causal variants (located in chromosome 16, Mbp positions 53.83 and 54.16), each explaining 0.5% of the simulated phenotype.

**II. Power-FDR performance in a simulation setting with heritability 0.5 and 50 causal variants**

**
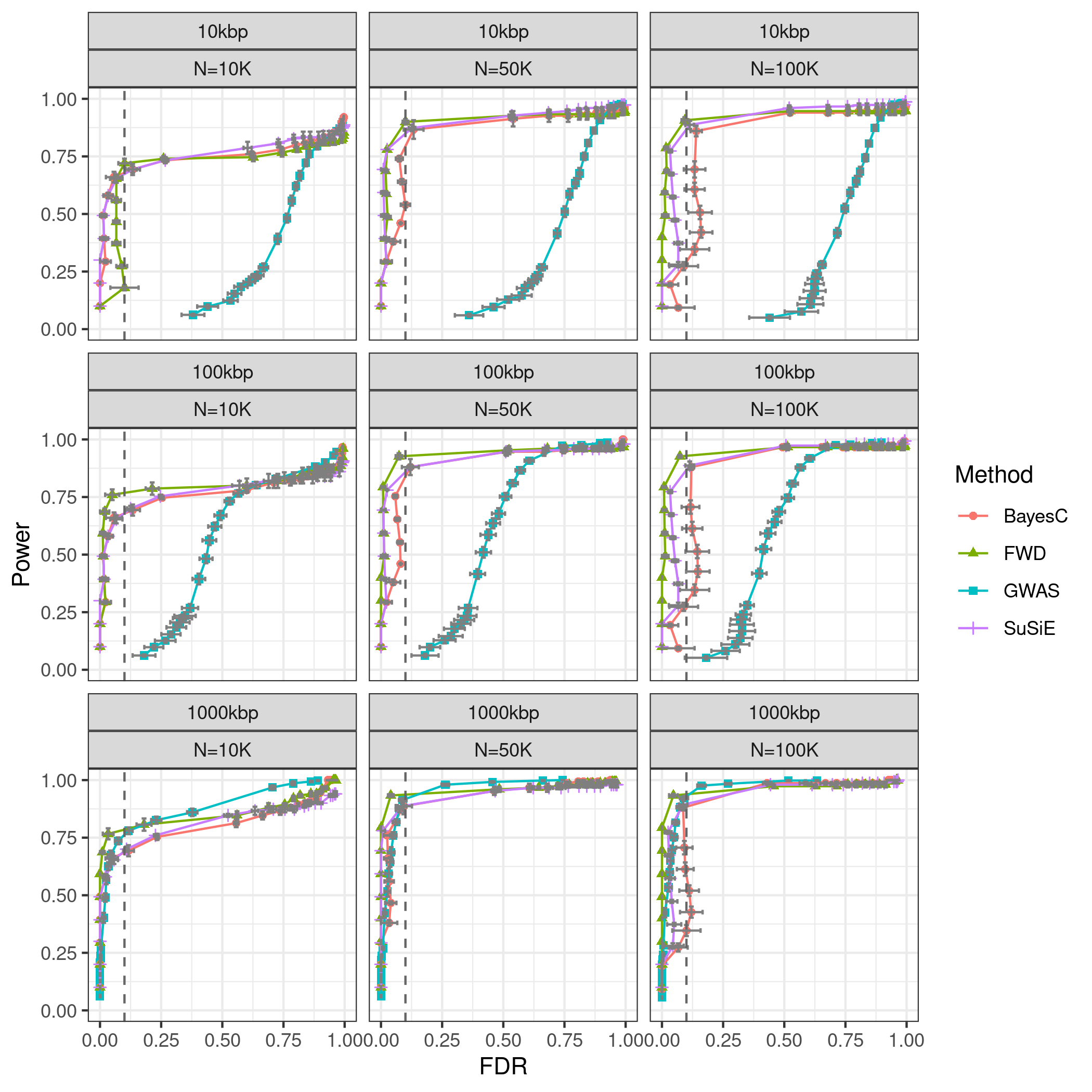
**

**Figure S4**: Power-FDR (False Discovery Rate) curves by sample size, mapping resolution, and statistical method used for the simulation scenario involving a heritability of 0.5 and 50 causal variants. For a mapping resolution of x-kbp, a SNP in a discovery set was considered a true discovery if its distance to the closest simulated causal variant was closer than x-kbp.

**III. Sample size and descriptive statistic by trait**

**Table S1**: Number of records and descriptive statistics by trait.

| Trait | # of Records | Mean | SD | Percentiles | | |
| --- | --- | --- | --- | --- | --- | --- |
|  |  |  |  | 0.025 | | 0.975 |
| Glucose | 266,708 | 5.12 | 1.21 | 3.86 | 7.65 | |
| Serum Urate | 291,376 | 5.70 | 0.26 | 5.15 | 6.18 | |
| Serum Creatinine | 291,594 | 4.26 | 0.20 | 3.89 | 4.65 | |
| LDL-Cholesterol | 291,223 | 3.57 | 0.87 | 2.00 | 5.39 | |
| HDL-Cholesterol | 266,887 | 0.34 | 0.26 | -0.16 | 0.85 | |
| Triglycerides | 291,508 | 0.42 | 0.51 | -0.50 | 1.49 | |

**Table S2:** Correlation (SE) between adjusted phenotypes and genomic prediction in a testing set (N=10,000) for three polygenic scores.

|  | SNP set and estimation method | | |
| --- | --- | --- | --- |
| Trait | GWAS-significant^a^ | | Whole-Genome |
|  | SMR ^b^ | BayesC ^c^ | BayesC (LBR) ^d^ |
| Glucose | 0.090 (0.010) | 0.115 (0.010) | 0.105 (0.010) |
| Serum Urate | 0.259 (0.010) | 0.340 (0.009) | 0.342 (0.009) |
| Serum Creatinine | 0.202 (0.010) | 0.279 (0.010) | 0.290 (0.010) |
| LDL-Cholesterol | 0.210 (0.010) | 0.309 (0.010) | 0.315 (0.010) |
| HDL-Cholesterol | 0.302 (0.010) | 0.415 (0.009) | 0.418 (0.009) |
| Triglycerides | 0.238 (0.010) | 0.342 (0.009) | 0.349 (0.009) |

a: SMR p-value <5e-8, b: Effects estimated through single-marker regression (SMR), c: Effects estimated by fitting all GWAS-significant SNPs using BayesC, d: Effects estimated by applying BayesC (using local Bayesian regressions) to all the available SNPs; only SNPs with posterior probability of inclusion >1/1,000 were used to compute the PRS.

**IV. Empirical FDR of decisions based on SMR p-values and on the Bayesian-FDR (BFDR), model BayesC**

**Figure S5:** Empirical False Discovery Rate (FDR) versus Bayesian FDR (BFDR, model BayesC) by sample size and mapping resolution (Kbp=Kilobase-pair). SNPs that cleared the BFDR threshold of the x- axis were considered discoveries; those that were at a distance greater than the mapping resolution on top of each plot were considered false discoveries.

**Figure S6:** Empirical False Discovery Rate (FDR) versus mapping resolution (Kbp: Kilobase-pair) by rejection rule for model Bayes C. BAYES 0.05 (0.10) considers a discovery all SNPs with BFDR <= 0.05 (0.10), SMR (5e-8) considers a discovery SNPs with single-marker-regression p-value <5e-8. Discoveries at a distance equal or smaller than the one indicated in the horizontal size were considered true discoveries, those at a higher distance were labeled as false discoveries.


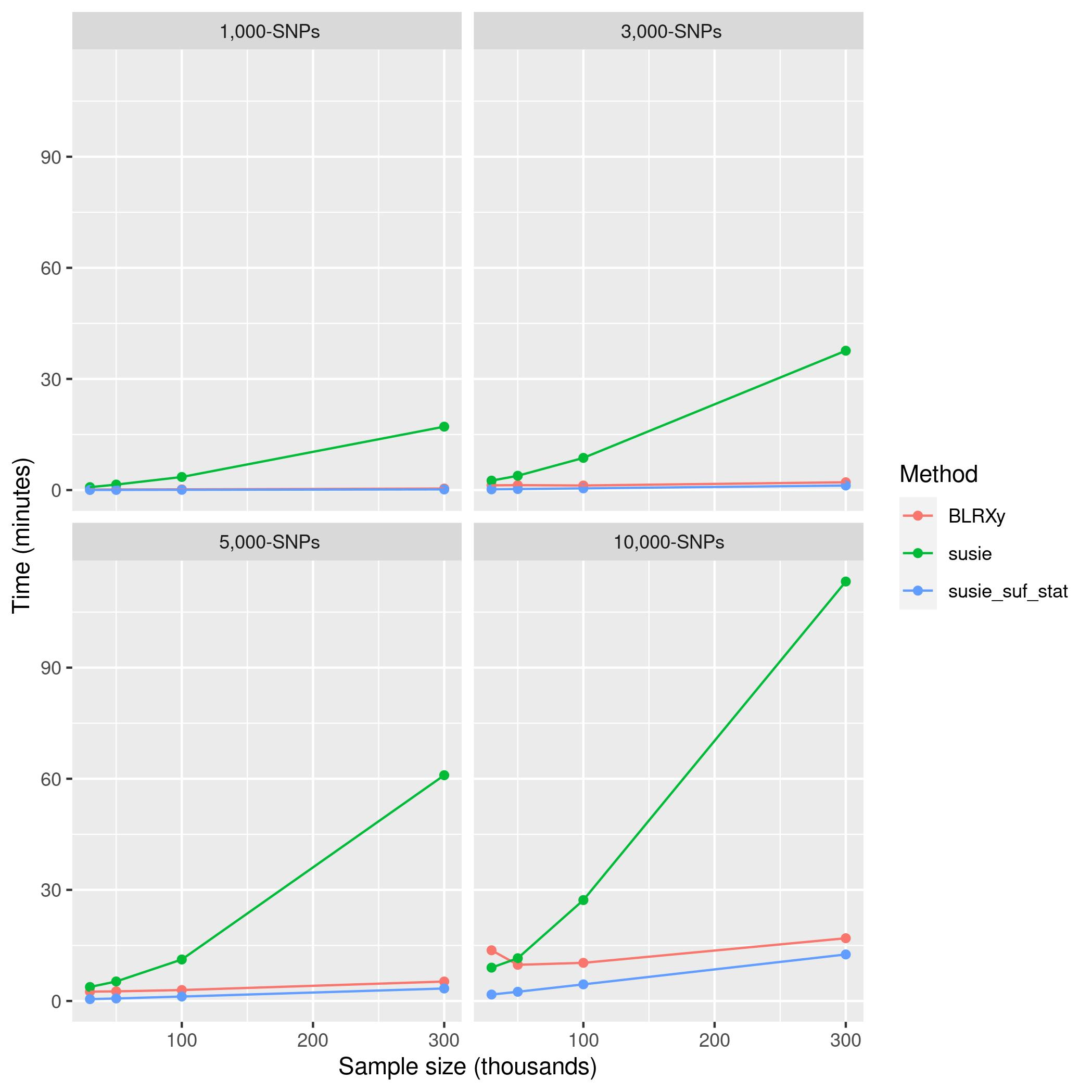


**Figure S7:** Average computing time by sample size (horizontal axis), number of SNPs in the model, and method. BLRXy() implements, among other models, the BayesC prior, and it is available in the BGLR R-package. SuSiE and SuSiE-sufficient statistics are available in the susieR R-package. For each scenario/method estimates were obtained fitting models to serum urate using 50 randomly chosen chromosome segments containing the number of SNPs specified in the panel. The same chromosome segments were used for all methods. The evaluation was performed in MSU’s High-Performance Computing Cluster, requesting nodes with at least 64GB of memory and a single core.

**IV. Supplementary Files**

**File S1**: Power-FDR by method, sample size, and mapping resolution, main simulation scenario (500 causal loci). [**File S1**](https://www.dropbox.com/s/tab0blx1lm4m8jw/FileS1.csv.gz?dl=0)

**File S2**: Power-FDR by method, sample size, and mapping resolution, simulation scenario with 50 causal loci. [**File S2**](https://www.dropbox.com/s/cexy97wi9llw4dd/POWER_FDR_IMPUTED_50.csv.gz?dl=0)
